# Geroderma Osteodysplastica With Concomitant Transposition of Great Vessels: A Case Report and Literature Review

**DOI:** 10.1155/crig/1397713

**Published:** 2024-11-21

**Authors:** Charbel Saad, Christine Aoun, Charbel Iskandar, Tony Hayek, Maroun Matar, Andre Megarbane

**Affiliations:** ^1^Department of Pediatrics, Lebanese American University Medical Center-Rizk Hospital, Achrafieh, Beirut, Lebanon; ^2^Department of Surgery, Division of Orthopedic Surgery and Trauma, Lebanese American University Medical Center-Rizk Hospital, Achrafieh, Beirut, Lebanon; ^3^Department of Human Genetics, Lebanese American University Medical Center-Rizk Hospital, Achrafieh, Beirut, Lebanon; ^4^Institut Jerome Lejeune, Paris, France

**Keywords:** Geroderma Osteodysplastica, osteoporosis, rare diseases, transposition of great vessels

## Abstract

Geroderma Osteodysplastica (GO) is a rare autosomal recessive connective tissue disease characterized by wrinkled skin and osteoporosis, two distinct aging-related features. A loss of function mutation in *GORAB* results in the disease. Immediately after birth, a cyanotic female neonate was found to have transposition of great vessels (TGV) that was corrected with an uneventful surgical recovery. The patient was noted to have wrinkled skin and hyperlaxity in her joints. After a complete nutritional and metabolic panel, in addition to karyotyping, imaging, skin histopathology analysis, and genetic testing she was found to have GO. We found two novel compound heterozygous mutations in *GORAB*: p.Asp236∗ and pAsp236Ala. This is the first study that reports the concurrent incidence of GO with TGV. The patient was started on bisphosphonates, which led to a reduction in the occurrence of fractures. An early diagnosis of GO is warranted to prevent or reduce bone density loss due to osteoporosis via initiation of bisphosphonate treatment. Whole exome sequencing remains the gold standard for diagnosing GO and ruling out phenotypically similar disorders.

## 1. Introduction

Geroderma Osteodysplastica (GO; OMIM#231070), is a rare autosomal recessive connective tissue disease initially described by Bamatter et al. in 1950 in five members of a Swiss family [1]. It is characterized by the presence of wrinkled, loose skin (more pronounced on the dorsum of the extremities) and characteristic facial features like saggy cheeks and excess skin under the chin, that give them a prematurely aged appearance, hyperextensible joints, osteoporosis, spinal deformities, fractures, and hip dislocation [2, 3].

GO is phenotypically similar to cutis laxa syndrome and genetic studies are the most accurate tests to correctly identify and distinguish between these different connective tissue diseases. GO results from a loss of function mutation in *GORAB* (Golgin, RAB6-interacting) which encodes for a Golgi-related transport protein [4]. The improvement in genetic studies resulted in the identification of new variants responsible for GO: compound heterozygous nonsense mutations in *GORAB* (NM_001320252.1; MIM∗607983).

We report the case of a compound heterozygote GO patient with concomitant transposition of great vessels.

## 2. Clinical Report

The propositus, a girl, was born at 38 weeks of gestation by spontaneous vaginal delivery. No known toxic exposures or unusual events were reported during the gestation, and fetal movements were normal. At birth, her weight was 3060 g, her length was 53 cm, and her head circumference was 37 cm. Directly after birth, she had perioral cyanosis, acrocyanosis, and severe respiratory distress unresponsive to oxygen administration which prompted neonatal intensive care unit admission. Physical exam showed hypotonicity of the upper and lower extremities as well as wrinkled skin, particularly on her abdomen and dorsa of her hands and feet. Cardiac auscultation revealed a continuous machine-like murmur characteristic of a patent ductus arteriosus heard at the suprasternal area. Femoral pulses were present as well as Moro, planning, and stepping reflexes. A trans-thoracic echocardiogram (TTE) revealed transposition of great vessels, a patent foramen ovale of 8 mm, and a patent ductus arteriosus of 3 mm. In addition, TTE revealed the presence of pulmonary hypertension with a pulmonary pressure of 50–60 mmHg. The patient underwent an arterial switch procedure the next day. Due to limited resource availability at that time, prostaglandin E2 was not given. The patient was placed on oral furosemide 10 mg and oral aldosterone 6.25 mg daily.

A karyotype and extensive metabolic panel came back normal and abdominal ultrasound and brain MRI showed normal and nonsignificant findings. A 4 mm skin punch biopsy was done for collagen typing. Collagen stains highlighted undulating collagen bundles unevenly distributed throughout the dermis and extending in the hypodermis. A pro-Collagen III level came back to be 8.67 U/mL (N 0.3–0.8). Histopathology of the specimen exhibited mild dermal fibrosis. The Verhoff–Van Gieson stain revealed a complete absence of elastic fiber network.

The baby girl was referred to our clinic at the age of 4 weeks for a diagnostic work-up of her dysmorphic features. At examination, length was 48 cm; weight was 2800 g; and her OFC was 34.2 cm (all below the third percentile). She was hypotonic, crying with a droopy face and maxillary hypoplasia.

**Figure 1:**
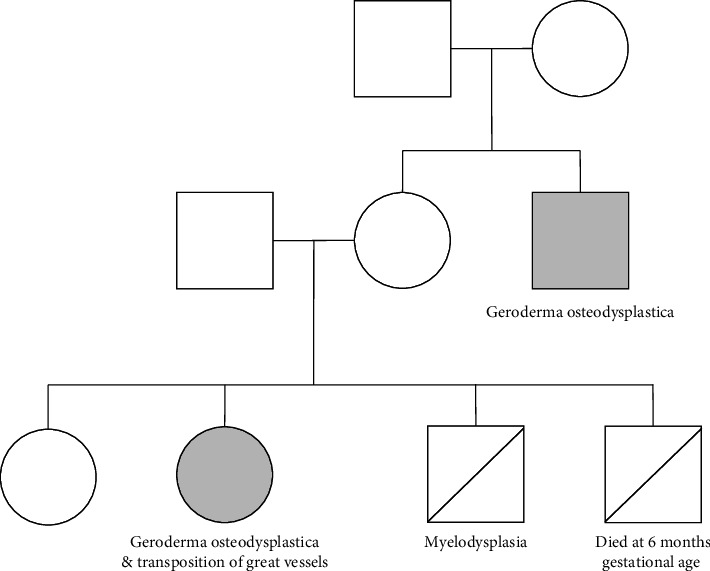
Family pedigree.

The patient has a healthy older sister and later dad a younger brother that died of myelodysplasia. She had another sibling that died at 6 months gestational age due to an unknown cause. There is no history of consanguinity with the parents, who are of Lebanese origin. A family pedigree is shown in Figure 1.

### 2.1. Genetic Studies

In view of the clinical picture, and after hearing that the patient has a third-degree relative who was recently diagnosed with GO, PCR amplification and bi-directional direct sequencing of exons one to five, and flanking intronic regions of *GORAB* (NM_152281) was done. Two pathogenic variants: c.630_631 insT (p.Asp211∗) and c.632A>C (p.Asp211Ala) in exon 4 of *GORAB* were found, both in a heterozygous state.

The variant c.705_706insT (p.Asp236∗) was found in a heterozygous state in the father and the mutation c.707A>C (pAsp236Ala) in a heterozygous state in the mother. According to Polyphen (1.0) and SIFT (0.0), the mutations are pathogenic. The patient was referred to orthopedics for bone maintenance and care and was started on 5 mg of alendronate per os daily in addition to Vitamin D supplementation.

During the next few years, the patient was seen regularly for routine clinical check-up. At 3 years of age, psychomotor assessment showed excellent learning and socialization skills. However, she showed a delay in milestones, particularly with her learning the ability to walk at 2.5 years of age. Grip and visual-manual coordination were of satisfactory quality and language milestones were reached on time. However, an insufficiency of tonic regulation was notable, in parallel with difficulties at the postural level and at the level of locomotion.

### 2.2. Osteoporosis, Bone Health, and Fracture History

**Figure 2:**
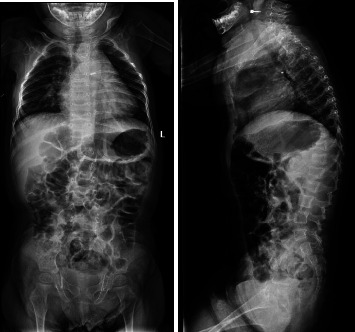
Radiographs at 1 year of age.

**Figure 3:**
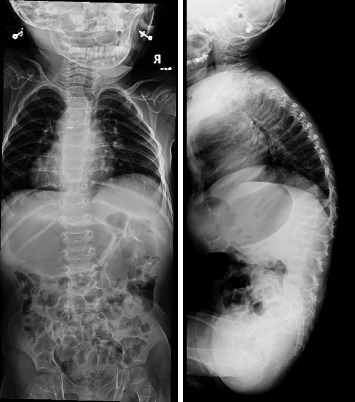
Radiographs at 1.75 years of age.

On a skeletal survey X-ray done at 15 days of age, there were no morphological abnormalities seen in the vertebral bodies, skull, or extremities. At 1 year of age, multiple thoracic bodies demonstrated biconcave endplates of a H-shaped deformity more prominent distally with anterior body height loss most prominent at T5 and L2 (Figure 2). After 9 months, a follow up radiograph showed an interval decrease in the height of the vertebral bodies, almost diffusely, more prominently at L3, L1, and L2, with wedging of the thoracic vertebral bodies mainly from T5-T12. There was levocurvature tilting of the thoracolumbar junction (Figure 3). At that time, the patient could not walk yet or stay standing up. In addition, talus valgus was noted in both legs in addition to pectus excavatum.

**Figure 4:**
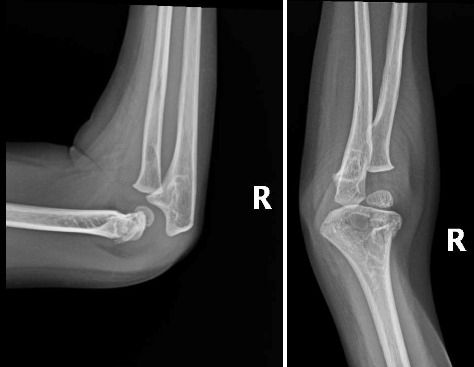
Radiographs of the right elbow.

**Figure 5:**
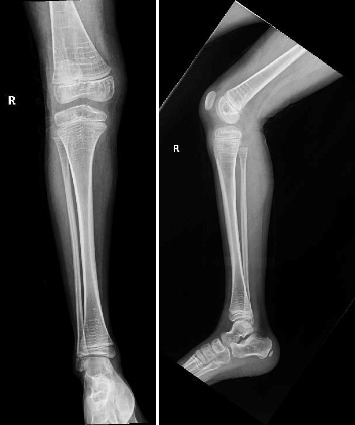
Radiograph of the legs at 6 years of age.

**Figure 6:**
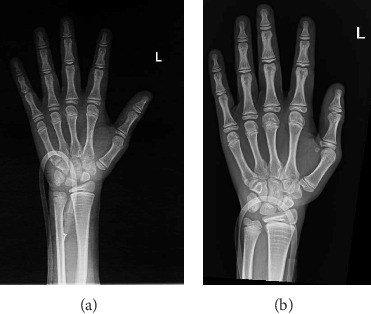
Hand radiographs for bone age assessment: (a) at 7 years 11 months chronological age, (b) at 9 years 3 months chronological age.

After a fall, the patient was found to have a supracondylar fracture in her right distal humerus with minimal posterior displacement and no evidence of dislocation (Figure 4). Assessment of the corticodiaphyseal index via radiograph of the legs (Figure 5) showed diffuse decrease in bone mineralization. Multiple metaphyseal lines are present, most probably corresponding to growth arrest lines associated with therapy. A bone age assessment via radiograph of the hand was done at 7 years and 11 months chronological age (Figure 6(a)) and showed an acceptable bone age of 7 years 10 months. However, a repeat assessment at 9 years 3 months showed a bone age of 11–12 years (Figure 6(b)).

## 3. Discussion

GO is very rare genetic disorder, with a prevalence so low that it has not been reported in the literature. Even though GO is a subtype of cutis laxa syndrome, it is not included in the autosomal recessive cutis laxa classification (Type I, II, III). GO has features similar to Type II cutis laxa (ARCL2A, ARCL2B) such as lax skin, prematurely aged features, hyperextensible joints with hypotonia, short height, and congenital hip dislocation [5]. However, the distinguishing features of GO from Type II cutis laxa include normal cognitive function, osteopenia resulting in pathological fractures, maxillary hypoplasia, and oblique creasing from the outer palpebral commissure to the lateral boundary of the supraorbital ridge [5]. GO exhibits similar characteristics to other syndromes as well, such as wrinkly skin syndrome [3] and certain bone fragility disorders [6]. However, GO is not allelic to wrinkly skin syndrome which is caused by mutations in *ATP6V0A2* [7]. A high index of suspicion for GO is imperative in patients presenting with skin and joint laxity, coupled with distinctive facial characteristics [2]. The clinical and radiological findings should lead to the diagnosis which should be confirmed by genetic testing. Nevertheless, accurately diagnosing GO is a challenge.  Table 1 shows genetic disorders that phenotypically overlap with GO.

The facies of GO patients are well described in the literature. As a result of the thin, laxed, and wrinkled skin, the eyelids and cheeks are droopy with a prominent and down-turned lower lip. These result in a sad and old-looking face. The head tends to be brachycephalic with a broad and prominent forehead and the maxillary and the malar bones are underdeveloped, resulting in a hypoplastic and flattened midface. Mandibular prognathism and Class I11 malocclusion, a high-arched palate, and dental crowding are also common. In a few instances, diminished height of the alveolar bone has been reported [8, 9].

Before her diagnosis of GO, our patient was diagnosed and treated for TGV. The literature has not reported any association between these two conditions or even any cardiac anomaly as a matter of fact [8]. In addition, it is still unknown whether an association exists between GO and the patient's family history of myelodysplastic syndrome.

The patient's delay in walking has been reported as a common finding in GO patients [9]. The patient's referral to a psychomotor showed no deficiency in cognitive abilities, which helped us distinguish her condition from Type II cutis lata. However, there were reports of mild intellectual disability in GO patients [8].

It is worthy to note that the patient's physical exam finding of pectus excavatum is surprising given the occasional association of GO with pectus carinatum [8]. However, the literature denotes an association between pectus excavatum and cardiac anomalies, and states that this condition can also be accentuated or even developed after cardiac surgery [10].

Prognosis in GO patients is widely related to the severity of osteoporosis. With most patients living quite normal lives, bisphosphonate treatment, along with Vitamin D supplementation, has been at the cornerstone of bone mineral density maintenance [6, 11]. In fact, the literature even reports the uncomplicated gestation of a GO patient [12]. To date, the patient is doing well; she is in good health and is going to a regular school.

## 4. Conclusion

In summary, this article reports the case of a compound heterozygote GO patient with concomitant TGV. The patient underwent surgical correction of the heart defect and was placed on bisphosphonate and Vitamin D treatment. Early diagnosis of GO via whole exome sequencing is essential in limiting bone resorption due to osteoporosis.

## Figures and Tables

**Table 1:** Genetic disorders overlapping with GO in phenotype.

Genetic disorder	Culprit gene	Inheritance	Location	Overlap with GO	Other manifestations
Cutis laxa Type IIA wrinkly skin syndrome∗	ATP6V0A2	AR	12q24.31	Loose and wrinkly skin connective tissue abnormalities	•Persistent wide fontanels
					•Frontal bossing
					•Slight oxycephaly
					•Downward-slanted palpebral fissures
					•Reversed-V eyebrows
					•Dental caries
					•Herniae
					•Foot deformities
					•Hip dislocations
					•Growth retardation

Ehlers–Danlos syndrome	COL5A1, COL5A2	AD	9q34.3, 2q32.2	Overlapping connective tissue abnormalities	•Skin hyperextensibility
					•Articular hypermobility
					•Tissue fragility
					•Loose-jointedness
					•Fragile, bruisable skin that heals with peculiar “cigarette-paper” scars

Wiedemann–Rautenstrauch syndrome	POLR3A	AR	10q22.3	Premature aging features	•Intrauterine growth retardation
					•Failure to thrive
					•Short stature
					•Progeroid appearance
					•Hypotonia
					•Variable mental impairment

Hutchinson–Gilford progeria syndrome	LMNA	AD	1q22	Premature aging osteolysis	•Short stature
					•Low body weight
					•Early loss of hair
					•Lipodystrophy
					•Decreased joint mobility
					•Osteolysis
					•Aged facial features

^∗^While both cutis laxa Type IIA and wrinkly skin syndrome share the same genetic mutation, WSS manifests with a less severe phenotype than cutis laxa Type IIA which has more systemic involvement and pervasive cutis laxa.

## Data Availability

The data that support the findings of this study are available from the corresponding author upon reasonable request.
